# A novel banana fiber pad for menstrual hygiene in India: a feasibility and acceptability study

**DOI:** 10.1186/s12905-021-01265-w

**Published:** 2021-03-26

**Authors:** Krishnashree Achuthan, Sharanya Muthupalani, Vysakh Kani Kolil, Anju Bist, Krishna Sreesuthan, Aswathy Sreedevi

**Affiliations:** 1 Amrita School of Engineering, Amrita Vishwa Vidyapeetham, Amritapuri, Kollam, 690 525 India; 2 School of Sustainable Development, Amrita Vishwa Vidyapeetham, Amritapuri, Kollam, 690 525 India; 3 Department of Community Medicine, Amrita Institute of Medical Sciences and Research Centre, Amrita Vishwa Vidyapeetham, Ernakulam, 682 041 India

**Keywords:** Sustainable development, Reusable and biodegradable, Menstrual hygiene management, Sanitary products, Menstrual health

## Abstract

**Background:**

Menstrual hygiene products used by women have evolved in the past several decades with comfort, ease of use and cost driving women’s choices. In a country like India, where women form nearly 50% of the population, the sheer volume of periodic menstrual non-biodegradable waste generated has significant environmental implications. With majority of the country hailing from low-middle class backgrounds, observing healthy menstrual hygiene practices with environmentally friendly products necessitates the consideration of affordable and highly sustainable alternatives. Further, during the COVID-19 pandemic, period poverty is higher than ever, causing women to turn to the reusable product market for affordable and long lasting alternatives. Hence, we studied the Feasibility and Acceptability (FA) of a novel banana fiber based menstrual pad (BFP) amongst women living in rural and urban environments.

**Methods:**

The quantitative study of FA of the BFP was conducted amongst 155 rural and 216 urban participants in India. For greater authenticity of the FA study, we considered participants who used BFP for more than 4 months (Rural = 111 and Urban = 186) in the study. The survey data included responses from participants from Bihar, Delhi, Karnataka, Kerala, Maharashtra, Tamil Nadu and West Bengal. A 22-item survey instrument was developed and validated using Exploratory Factor Analysis (EFA) and reliability test (Cronback’s $\alpha$). Binomial logistic regression analysis was used to analyse the factors that affect the FA of BFP based on the survey responses. In addition to survey analysis, environmental sustainability through $\hbox {CO}_2$ footprint analysis, microbial load, pH and the ability of the BFP to withstand pressure after absorption were also studied.

**Results:**

The results indicated high levels of feasibility (rural $= 82.2\%$, urban $= 80.3\%$ and acceptability (rural $= 80.2\%$, urban $= 77.5\%$) of BFPs across both participant groups. Comparing key BFP characteristics such as leakage and comfort to participants’ prior practices revealed general satisfaction on the performance of BFP, leading to them recommending BFPs to others. User perception on the reasons for their preference of BFP highlighted their concern for environment, health and cost as decisive factors. The microbial load on a 3 year reused BFP was found to be similar to an unused BFP. Regression analysis showed cost as an important indicator for feasibility ($\hbox {OR} =1.233$; 95% CI = 1.083–3.248) and acceptability ($\hbox {OR}= 1.422$; 95% CI = 1.203–3.748) amongst rural participants.

**Conclusion:**

Based on feasibility and acceptability results, BFP is a promising consideration as an environmentally sound, non-invasive; yet reusable alternative to fulfil MHM needs in populous countries such as India. Longer term studies in larger samples are necessary to validate these findings.

**Supplementary Information:**

The online version supplementary material available at 10.1186/s12905-021-01265-w.

## Background

It is well recognized that poor Menstrual Hygiene Management (MHM) practices adversely affect the initiatives of countries toward achieving a number of important sustainable development goals i.e. SDGs 2, 3, 4, 5, 6, 8, and 12 [1–3]. The wide spectrum of challenges are associated with both women’s menstrual health needs and those of the environment. These include access to clean absorbent materials; availability of safe, and private spaces for cleaning, changing; disposal of materials; access to adequate menstrual and reproductive health education; and socio-cultural norms that stigmatize menstruation and limit social support [4–7]. More recently, poor MHM has been recognised as a global public health problem, resulting in a multi-sectorial response [8].

Amongst 336 million women of reproductive age in India, 22% live in rural areas. Due to the prevalence of poverty, many women resort to unhygienic menstrual practices such as using old clothes [9, 10] to manage their cycles. These render women vulnerable to health risks and infections [11–13]. Several reports also confirm unavailability of better and affordable alternatives as primary causes for adolescents to drop out of schools and women to have unmet needs [14–20]. During the COVID-19 pandemic, when essential services were suspended, poverty and deprivation of menstrual materials affected thousands of girls and women [21].

The Government has made significant strides in increasing women’s access to MHM resources [22] through distribution of subsidized disposable sanitary products [18] leading to their increased adoption [8, 23]. Although this was intended to foster proper MHM practices, women are unaware of the resultant environmental pollution [18, 24, 25]. Every year, discarded sanitary napkins alone generate 113,000 tonnes of menstrual waste in India [26]. In rural areas, the options for disposing menstrual waste include burying, burning, and throwing them into pit latrines. In contact with soil, disposable napkins kill the soil’s microflora and delay decomposition [9, 23]. Recycling is crucial to responsible waste processing. However, recycling disposable sanitary products is highly complex as sanitation systems are not equipped to cope with menstrual waste [26]

A possible solution to both problems i.e. provide more hygienic alternatives to MHM while causing minimal impact to the environment is to explore reusable, sustainable and greener alternatives.The future of fostering good MHM practices lies in scaling and creating affordable alternatives that are both safe and largely biodegradable [27]. Additionally having such options are critical in times such as COVID-19 with reduced access and exacerbated vulnerabilities [28]. Reusable cloth, used by rural women, are limited in their absorptive capacity for typical durations of use and are perceived unhygienic. On the other hand, reusable menstrual cups’ necessity for insertion has dampened its adoption [4]. Reusable materials that are culturally acceptable and sustainable are more appropriate [29]. Although natural fiber based pads positively aligns to these requirements, women lack exposure and information due to lack of in-depth studies [1]. Studies are needed to conduct rigorous assessment of risks [30] on feminine hygiene products. Further, feasibility studies are necessary to understand women’s preferences. Prior studies on menstrual absorbents have lacked sufficient engagement from the prime stakeholders i.e. women themselves related to their perceptions, needs and choices, and correlating their preferences to their social, economic and geographical contexts [31]. In particular, the opinions and experiences of rural women in remote areas are under reported [32, 33].

The objectives of this paper are to explore the feasibility and acceptability of a reusable, banana fibre based pad (BFP) used by rural and urban participants, as a means to meet menstrual health outcomes and target environmental sustainability. Our contribution includes: (1) developing an instrument to gauge feasibility and acceptability of BFPs followed by its validation, (2) assessing BFP performance by capturing usage experience of rural and urban participants that is statistically analyzed and (3) exploring the carbon footprint of disposable pads and BFPs and (4) preliminary characterizing of BFP microbial load as per BIS guidelines [34].

## Methods

### Description of measures

A market ready product, BFP, was chosen [35] to test the feasibility and acceptability amongst women. A cross-sectional study was conducted with a validated 22-item instrument (see Additional file 1).

The operational definition for feasibility included assessing the ability of BFPs to meet end user needs, its maintenance, safety, affordability and environmental sustainability through: (1) seven items in the survey instrument that related to the user’s direct experience of leakage, duration of use, place of usage, washing and drying of pads (Table 1), (2) theoretical computations on carbon footprint to determine the environmental sustainability and (3) microbial load, pH test and pressure squeeze test on BFP samples.

Acceptability was determined from user satisfaction, BFP’s adequacy at being the primary choice for users and recommended choice for others as well as motivational causes for adoption. This was assessed by: eight questions in the survey instrument on comfort, ease of use, ease of maintenance, their likelihood to continue using as well as recommending to others and the primary reasons for both (Table 1).

**Fig. 1:**
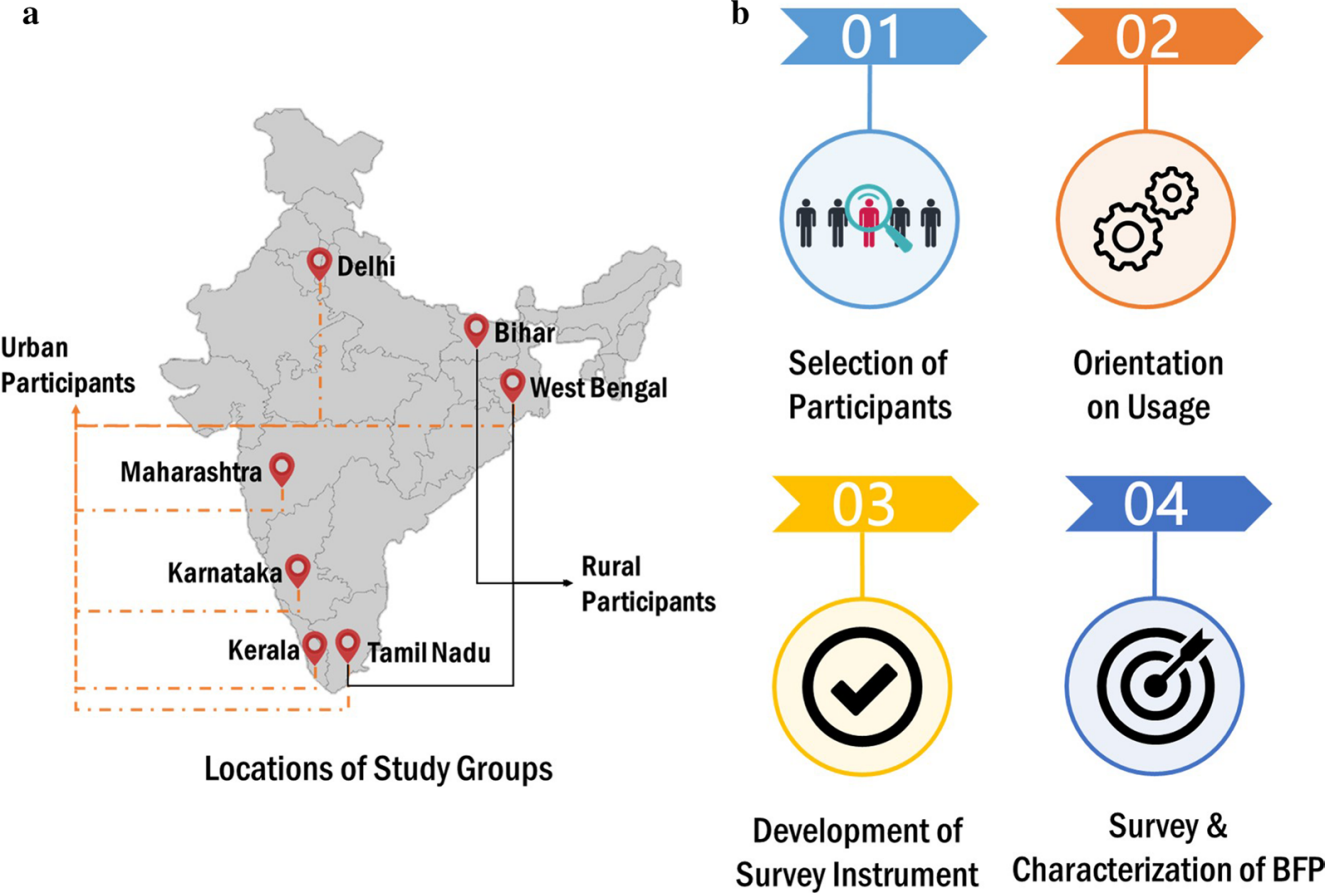
**a** The locations in India covered under the study; **b** The various steps of BFP study

**Fig. 2:**
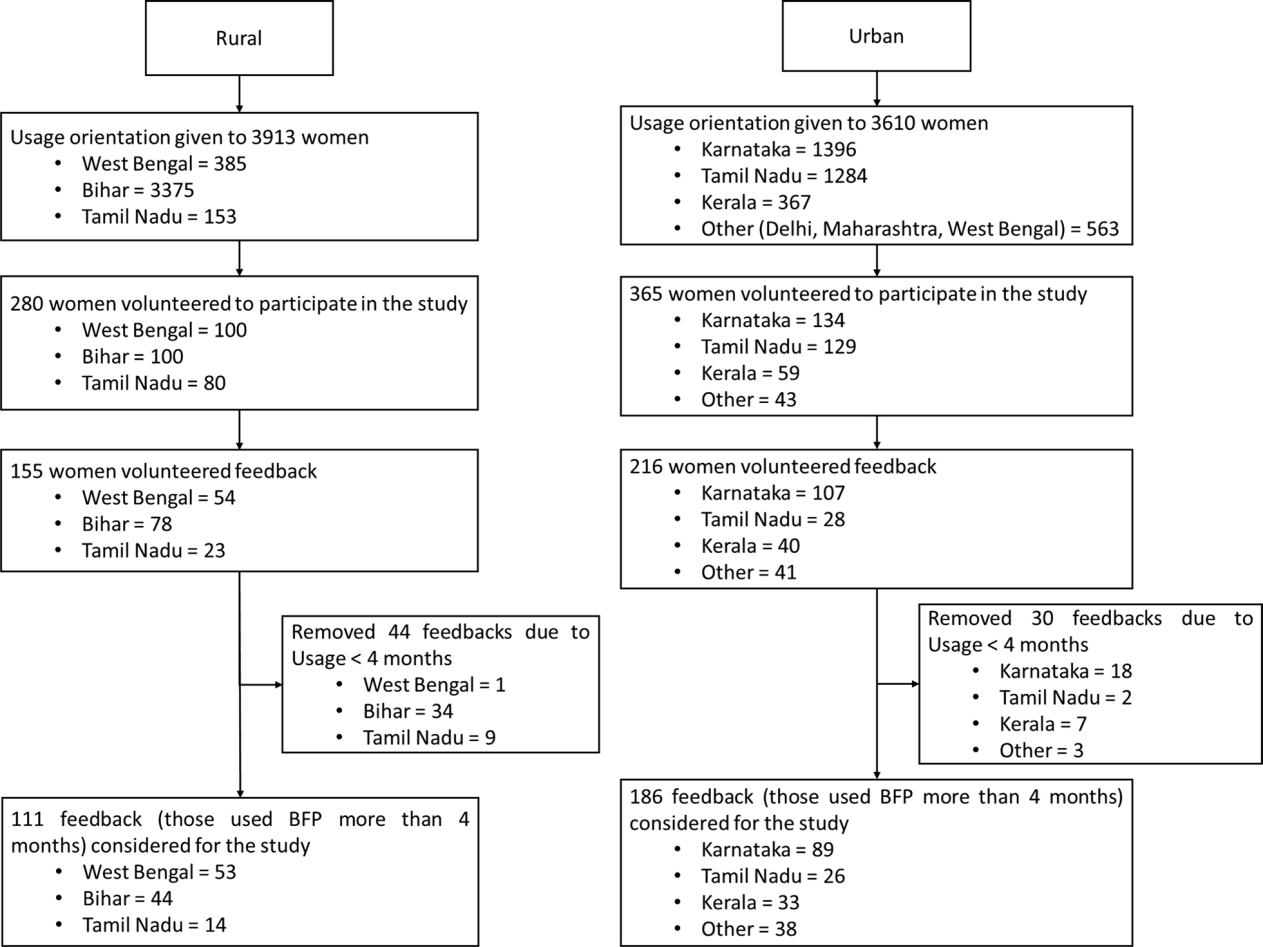
Selection of participants

**Table 1:** Survey Instrument and its validity

No.	Item	Factor I: Feasibility	Factor II: Acceptability
Q8	Preferred place of usage	**.708**	− .161
Q9	Experience with leakage	**.612**	− .219
Q10	Duration of single pad usage	**.627**	− .159
Q11	Duration of wash	**.703**	− .187
Q12	Method of wash	**.715**	− .106
Q13	Duration taken to dry	**.648**	− .081
Q14	Method of dry	**.687**	− .173
Q15	Preference of sanitary products	− .247	**.837**
Q16	Recommending BFP to others	− .233	**.858**
Q17	Comfort and ease of use and reuse	− .244	**.826**
Q18	Ease of washing and cleaning	− .239	**.816**
Q19	Others in the family wanting to switch	− .213	**.821**
Q20	Like to continue	− .111	**.723**
Q21	Reason for preferring BFP	− .041	**.740**
Q22	Reason for recommending BFP	− .209	**.840**
	# items	7	8
	Item variance	1.28	2.00
	Cronbach’s Alpha ($\alpha$)	0.817	0.937

Values in the bold (values above 0.5) are representing those that conform to that category

### Participants

Study participants were recruited from diverse backgrounds and living environments across seven states in India via partnership with a non-governmental organization [36–38]. Participants were broadly classified as living in rural or urban environments (Fig. 1).

Rural participants hailed from eight villages in three states i.e. West Bengal, Bihar and Tamilnadu. All women in these villages (i.e. a total of 3913 females of age 10–45) were oriented on usage of BFPs and menstrual hygiene. Amongst them, 280 females volunteered to participate in this study. Of these participants, 155 volunteered to provide detailed feedback as shown in Fig. 2.

In urban cities that are in Karnataka (i.e. Bangalore, Mangalore), Tamilnadu (i.e. Chennai, Coimbatore), Kerala (i.e. Thiruvananthapuram, Kochi, Thrissur) and a few other cities such as Delhi and Mumbai. usage orientation was conducted for 3610 females. Amongst them, 365 females volunteered to participate in the study. Of these participants, 216 volunteered to provide feedback. All the participants gave informed consent that indicated their willingness to participate in the study with their understanding that they could withdraw at any point.

The survey instrument was administered to those who were using BFP at that time and had used it for at least 4 months. This reduced the total sample size to 111 amongst rural participants and 186 amongst urban participants. The minimum sample size ($\hbox {N} = 111$) to compare two groups with 5% precision was identified using the equation ($\hbox {N} = ((\hbox {Z}_{\alpha /2}+\hbox {Z}_\beta )^2\times 2\times \sigma ^2) / \hbox {d}^2$) [39]. To verify the microbial load, samples of used BFPs were taken from users who had been using them for more than 2 years. One such sample collected from urban users had been used for 3 years (or 36 cycles).

### Instrument validity

The construct validity of the instrument was evaluated though Exploratory Factor analysis (EFA). EFA was done (n = 300) using K1 rule (eigen value $> 1$), scree plot analysis, parallel analysis, and varimax rotation for the survey responses [40–42]. This resulted in extraction of two factors i.e. feasibility and acceptability based on their 59.4% contribution to the total variance. Kaiser–Mayer–Olkin (KMO) test was run for sample adequacy and the value was 0.944 for the 22-item sample. The Bartlett’s test statistic (2410.604 at $\textit{p} < 0.001$) being higher than the critical value confirmed the homogeneity of variance. Factor structure and loadings are presented in Table 1. Content validity was maintained by selecting items with factor loadings $>0.50$. The content validity of the instrument was verified by public health medical personnel with over a decade of field experience. The reliability coefficient Cronbach’s $\alpha$ for feasibility was 0.817 (7 items) and for acceptability was 0.937 (8 items). The overall reliability coefficient was found to be 0.700. The inter-correlation between the feasibility and acceptability sub scales was found to be negligent, confirming independence of the respective subscales.

**Table 2:** Socio-demographic characteristics of study participants

Item	Rural ($$\hbox {N} = 111$$)		Urban ($$\hbox {N} = 186$$)		Significant test
	Frequency	Percentage	Frequency	Percentage	$$\chi ^2$$, *df*, *p*
Age					
10–19 years	78	70.3	56	30.1	47.39, 3, 0.000*
20–25 years	13	11.7	33	17.7	
$$>\,25$$ years	19	17.1	95	51.1	
No response	1	0.9	2	1.1	
Education					
Lower primary school	1	0.9	–	–	131.41, 5, 0.000*
Upper primary school	6	5.4	–	–	
High school	46	41.4	55	29.6	
Pre-degree/higher secondary school	8	7.2	–	–	
College	13	11.7	129	69.4	
No response	37	33.3	2	1.1	
Profession					
Working professional	–	–	78	41.9	162.66, 4, 0.000*
Student	20	18.0	88	47.3	
Homemaker	59	53.2	15	8.1	
Farmer	27	24.3	1	0.5	
No response	5	4.5	4	2.2	
Prior pad choices					
Disposable non-biodegradable	61	55.0	110	59.1	38.91, 3, 0.000*
Reusable non-biodegradable	–	–	2	1.1	
Reusable biodegradable	25	22.5	3	1.6	
Hybrid usage of various sanitary products	25	22.5	71	38.2	
First person in the family to to make switch					
Yes	107	95.5	166	89.2	3.17, 1, 0.075
No	5	4.5	19	10.2	
Money spent every month on sanitary products					
More than ₹100 ($US 1.31)	–	–	47	25.3	103.58, 4, 0.000*
₹50–₹100 ($US 0.65–$US 1.31)	11	9.9	54	29.0	
Less than ₹50 ($US 0.65)	67	60.4	74	39.8	
Using cloth pad and no expense	33	29.7	1	0.5	
No response/Don’t know	–		10	5.4	

*Significant at $$\textit{p} < 0.05$$

### Data collection

The socio-economic and demography details (Table 2) of the participants were captured through seven questions in the instrument. From the responses received, between 71.6 and 87.5% of women from both urban and rural participants had used BFP for more than 4 months. 21% of urban respondents had used BFP between 1 and 3 years. Data was collected from these participants using the validated survey instrument which was developed in English and translated into four regional languages (Hindi, Bengali, Tamil, Malayalam). Assistance from community health workers enabled conduct of in-person interviews. Their trusted reputation among the community and familiarity with local women encouraged openness in responses. An online survey form of the instrument in English was used to collect the feedback from the urban participants.

### Usage orientation

Usage orientation workshops conducted in participants’ neighbourhoods included two key aspects. The first aspect was an educational presentation on choices of sanitary pads and their impact on the environment. Common menstrual health issues were also presented. The second aspect detailed the methods to maintain hygienic practices, practical suggestions on the frequency and duration of usage of BFPs, its maintenance and disposal. Knowledge transfer from women who have used BFPs for several years to participants took place in the form of videos and interactive sessions too. In a comfortable setting, there was open dialogue about individual variations in menstruation, navigating privacy in school or at work. The talks were conducted in local languages with the support of health workers and medical professionals. Further, participants were taught how to wash and dry the BFP to maintain optimal quality. This included generic tips on prevention of tears and stains, tools for scrubbing, and places of drying. In alignment with past works, [43] the workshops were a new experience for most women who had no access to information on MHM as well as knowledge on the origin or destiny of disposable pads. The women procured BFPs either online [44] or through the NGO’s (non-governmental organization) distribution centers in over 20 states with stocked reserves of BFPs. Continuous support on BFP use and maintenance was provided through the health care workers for rural participants and via email and social media to urban users.

### Data analysis

Descriptive statistics were calculated in the form of frequencies and percentages. A significant test ($\chi ^2$ test analysis) was conducted to analyse the difference between the two study groups (rural and urban). The responses for each of the fifteen variables for feasibility and acceptability were given three possible scores (0, 0.5 or 1). A fully positive response was rated at 1, partially positive at 0.5 and fully negative response at 0. The total possible score for feasibility was 7 and that for acceptability was 8. By averaging the responses from each of the variables and their sum totals, we derived final scores for feasibility and acceptability. Binomial logistic regression analysis was conducted to identify factors that affected the feasibility and acceptability (FA) of BFP. The socio-demographic variables (age, education, profession, and amount of money spent every month on sanitary product) were selected as the independent variables and the feasibility and acceptability of BFP were selected as the dependent variables. The above average score ($\ge 3.5$) of feasibility is converted into ‘high feasibility’ and below average score ($< 3.5$) is converted to ‘low feasibility’ for the binomial logistic regression analysis. Similarly, the score of acceptability is converted into ‘high acceptability (score $\ge 4$)’ and ‘low acceptability (score $< 4$)’. The results were computed at 95% confidence interval (CI). The analysis was conducted in IBM SPSS V.20 software and no adjustment was made for multiplicity. Multicollinearity of the independent variables based on the variance inflation factor (VIF) was found to be moderate i.e. between 1 and 4 [45, 46].

## Results

This study is the first of its kind to assess the user perception of a non-intrusive, natural fiber based alternative such as BFP through characterization of feasibility and acceptability (FA). Centering the FA study around those that would benefit the most from MHM i.e. rural women and comparing that to urban women who have several potential choices is one of the unique contributions of this study.

Socio-demographic details of participants are detailed in Table 2. Rural participants ranged from 10 to 40 years of age, with 70.3% between 10 and 19 years, 11.7% between 20 and 25 years, and 17.1% over 25. Approximately 41.4% had 8 years of schooling and were educated through high school, 6.3% did not study beyond primary school, nearly 7.2% attended school beyond secondary school, and 11.7% had completed college education. Approximately 53.2% were homemakers, nearly 24.3% were farmers, and 18% were students (Table 2).

Amongst urban women 30.1% were 10–19 years old, 17.7% were between 20 to 25, and 51.1% were above 25 years of age. Nearly one-third of urban participants completed high school and 69.4% had completed college. Almost half were either college or school students, while 41.9% were working professionals, and 8.1% were homemakers.

More than half of the participants in rural (55.0%) and urban (59.1%) participants indicated that they used disposable and non-biodegradable pads while approximately 22.5% of rural girls and women had used reusable biodegradable (cotton cloth) in their prior practice. Almost 95.5% rural and 89.2% urban participants were first in their households to try BFP.

The $\chi ^2$ test indicated that both rural and urban groups differed significantly from each other in socio-demographic characteristics such as age ($\chi ^2$: 47.39; *df*: 3; *p*: 0.000), education ($\chi ^2$: 131.41; *df*: 5; *p*: 0.000), profession ($\chi ^2$: 162.66; *df*: 4; *p*: 0.000), prior pad choices ($\chi ^2$: 38.91; *df*: 3; *p*: 0.000) and money spent every month on sanitary products ($\chi ^2$: 107.72; *df*: 4; *p*: 0.000).

**Table 3:** Responses of the participants on the feasibility of the BFP

Item	Responses	Rural (N = 111) Frequency (%)	Urban (N = 186) Frequency (%)
Preferred place of usage	Any time	84 (75.7)	91 (48.9)
	At work/college/school	–	12 (6.5)
	At home only	20 (18.0)	80 (43.0)
	At night only	5 (4.5)	24 (12.9)
Experience withleakage	Same as disposable pads	7 (6.3)	34 (18.3)
	Less leakage than disposable pads	54 (48.6)	65 (34.9)
	More leakage than disposable pads	–	22 (11.8)
	No leakage at all	50 (45.0)	65 (34.9)
Duration of single pad usage	Less than 3 h	9 (8.1)	8 (4.3)
	3–4 h	30 (27.0)	56 (30.1)
	5–6 h	68 (61.3)	84 (45.2)
	More than 6 h	4 (3.6)	37 (19.9)
	No response	–	1 (0.5)
Duration of wash	Less than 1 min	1 (0.9)	3$^a$ (3.0)
	1–2 min	4 (3.6)	28$^a$ (28.0)
	More than 2 min	105 (94.6)	69$^a$ (69.0)
Method of washing	Brush wash	11 (9.9)	7 (3.8)
	Soak first in water and then wash by hands	95 (85.6)	174 (93.5)
	Machine wash	–	4 (2.2)
	Stone wash and later soak in disinfectant water for sometime	–	1 (0.5)
	No response	5 (4.5)	–
Duration taken to dry pads	1–3 h	27 (24.3)	64 (34.4)
	4–6 h	61 (55.0)	62 (33.3)
	More than 6 h	7 (6.3)	26 (14.0)
	One day	14 (12.6)	28 (15.1)
	Don’t know	2 (1.8)	6 (3.2)
Method of drying pads	In my room/bathroom	12 (10.8)	44 (23.7)
	In the open with other clothes, but I cover it with a towel	10 (9.0)	13 (7.0)
	In the open far from rest of the cloths	88 (79.3)	126 (67.7)
	In the drying machine	–	1 (0.5)
	No response	1 (0.9)	2 (1.1)

^a^Missing data 86

### Feasibility of BFP

Feasibility was assessed by the survey instrument (Table 3) and supplemented by carbon emissions estimations and preliminary analytical tests on BFP. Investigating whether the usage patterns depended on location, activity, or time of day, between 49 and 76% of urban and rural participants reported that they use BFP at all times. However, 43% of urban participants indicated they preferred to use BFP at night only.

With respect to duration of use of a single BFP, between 75 and 88% of rural and urban women indicated they used one pad for 3 to 6 h before changing. When asked about leakage, the overwhelming response from rural women was that BFPs either did not cause any leakage (45%) or had less leakage than the disposable pads (48.6%). The responses from urban participants indicated 70% experiencing either less leakage or no leakage at all, 18.3% experiencing no difference in leakage amounts and 11.8% experiencing more leakage than when they used disposable pads.

Methods of washing and drying are important aspects of BFP maintenance. A majority of rural (95%) and urban (69%) women indicated they took more than 2 min to wash BFPs. Most of them (i.e 85.6% urban and 93.5% of rural women) reported that they soak BFPs before washing by hand. Such care helps increase the longevity of BFPs. In terms of drying, while 34.4% or urban users reported that BFPs dried within 3 h, a majority (55%) of rural users and 33.3% of urban users said it took 4–6 h. Approximately 20% of rural women experienced longer drying times. Up to 79.3% of rural users and 67.7% of urban users said they dried their pads away from other laundry. Up to 24% of urban women reported drying their pads in their bathroom, without direct exposure to sunlight. Long-term users of BFP (i.e., used for over a year) were asked for their observations regarding physical changes to the pad over time, including all types of degradation in the pad. Almost 59% of rural users and almost 48% of urban users indicated that even after a long period of use, the pad showed little or no degradation, while 10% of users reported incidents of leakage occurring more often after one year of use.

#### Environmental sustainability

**Fig. 3:**
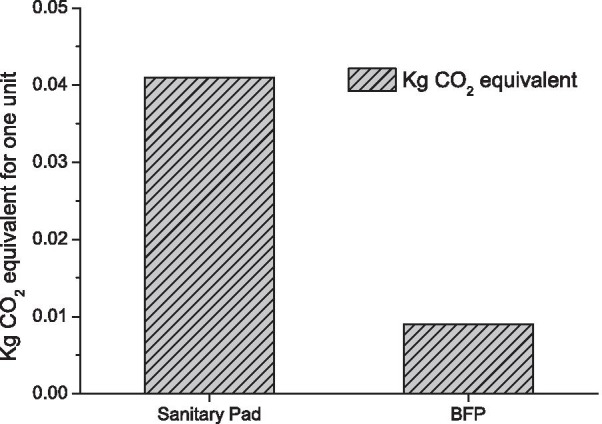
${\hbox {CO}}_2$ equivalent (kg) of one unit BFP and Disposable sanitary pad

The BFP is reusable and largely biodegradable. Since banana plants are monocarpic, replantable and their pseudo-stem is an agricultural waste product, banana fiber is one of the most sustainable raw materials for sanitary pads. They are easy to mass produce and energy-efficient [33, 47, 48]. The Fig. 3 shows the approximate kg ${\hbox {CO}}_2$ emissions from one unit of disposable sanitary pad and BFP. The approximate ${\mathrm{CO}}_2$ emission of one disposable sanitary pad is 0.041 kg ${\hbox {CO}}_2$ [49], while that of one unit of BFP, is expected to be far less than 0.01 kg ${\hbox {CO}}_2$.

**Fig. 4:**
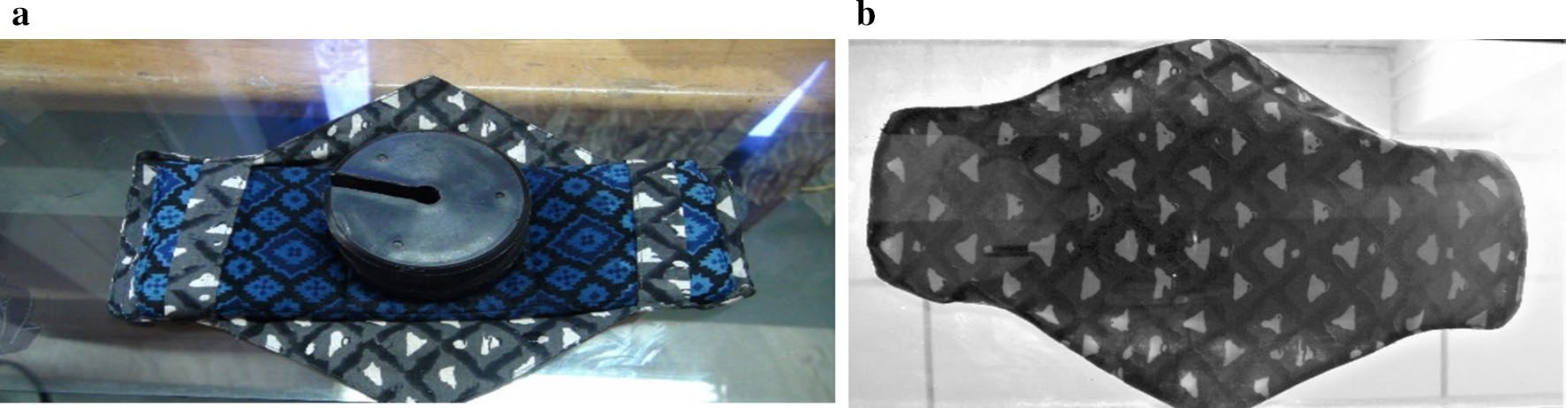
1 kg weight on reusable pad after pouring 30 mL of coloured distilled water: **a** top view, **b** bottom view

### Microbial load tests and pH

A preliminary study on the microbial load of the BFP was characterized using the standardized bioburden test. Conclusive evidence from prior studies indicate certain diseases such as toxic shock syndrome and candidiasis, are caused by microbes such as *S. aureus* [50] and *Candida albicans* [51]. Sample specimens taken from new BFPs were compared to heavily reused BFPs (over 3 years or 36 cycles) for the quantity of *Staphylococcus aureus*, (*S. aureus*), *C. albicans* (*C. albicans*) and other pathogens. It was observed that 22 mL and 17 mL of 0.9% saline was retained in the 5 g of both samples respectively. The total microbial load in both samples was comparable to each other and was less than 1000 CFU/mL. This affirms the safety of the BFP as there was no significant increase in the microbial load.

The pH testing was found to be between the range of 6–8.5, which is the optimum pH for sanitary products [52]. Within this range of pH, microbial activity is not encouraged, making BFPs safe for women to use.

#### Ability to withstand pressure after absorption

Women expect menstrual absorbents to be reliable and prevent soiling [43]. To test the BFP for leakage, as per the BIS guidelines [34], 30 mL of water was poured at a controlled rate onto the surface of the pad. One kg weight was then applied on the surface in order to squeeze the water out in all directions. Figure 4 shows the top and bottom views of a BFP subject to pressure squeeze test, confirming no leakage under testing conditions.

**Table 4:** Responses of the participants on the acceptability of the BFP

Item	Responses	Rural (N = 111) Frequency (%)	Urban (N = 186) Frequency (%)
Preference of sanitary products	Both disposable pads and BFP	38 (34.2)	97 (52.2)
	BFP 80% of the time	–	38 (20.4)
	Menstrual cup and BFP	–	5 (2.7)
	Only BFP	69 (62.2)	42 (22.6)
	BFP and another brand of reusable pads	–	2 (1.1)
	No response	4 (3.6)	2 (1.1)
Recommending BFP to others	Very likely	2$^{\mathrm{a}}$ (14.3)	104$^{\mathrm{b}}$ (78.2)
	Likely	9$^{\mathrm{a}}$ (64.3)	20$^{\mathrm{b}}$ (15.0)
	Unlikely	3$^{\mathrm{a}}$ (21.4)	2$^{\mathrm{b}}$ (1.5)
	Very unlikely	–	7$^b$ (5.3)
Comfort and ease of use and reuse	Strongly agree	104 (93.7)	103 (55.4)
	Agree	3 (2.7)	53 (28.5)
	Neutral	–	20 (10.8)
	Disagree	4 (3.6)	9 (4.8)
	Strongly disagree	–	1 (0.5)
Ease of washing and cleaning	Strongly agree	106 (95.5)	95 (51.1)
	Agree	3 (2.7)	61 (32.8)
	Neutral	–	16 (8.6)
	Disagree	–	4 (2.2)
	Strongly disagree	2 (1.8)	10 (5.4)
Are others in the family now wantingto make switch to BFP?	Yes	67 (60.4)	85 (45.7)
	No	29 (26.1)	62 (33.3)
	Don’t know	15 (13.5)	39 (21.0)
Like to continue to use the BFP?	Very likely	98 (88.3)	74$^c$ (74.0)
	Likely	13 (11.7)	19$^{\mathrm{c}}$ (19.0)
	Unlikely	–	2$^{\mathrm{c}}$ (2.0)
	Very Unlikely	–	5$^c$ (5.0)
Reason for preferring BFP?	Easy to handle	78 (70.3)	62 (33.3)
	Absorbs better	74 (66.7)	63 (33.9)
	Feels clean	10 (9.0)	48 (25.8)
	Economical	82 (73.9)	120 (64.5)
	Eco-friendliness	97 (87.4)	165 (76.6)
	I feel traditional	4 (3.6)	25 (13.4)
	I feel modern	8 (7.2)	14 (7.5)
Reason for recommending BFP?	Concern for the environment	87 (78.0)	120 (64.5)
	Concern for the health	71 (63.6)	34 (18.4)
	Money spent every month	58 (52.3)	31 (16.4)
	No response	9 (8.3)	1 (0.7)

Missing data ^a^97; ^b^53; ^c^86

**Table 5:** Scores and percentages of feasibility and acceptability of BFP

Feasibility					Acceptability			
	Rural		Urban		Rural		Urban	
	$< 4 \,\hbox {months}$	$> 4 \,\hbox {months}$	$< 4 \,\hbox {months}$	$> 4 \,\hbox {months}$	$< 4 \,\hbox {months}$	$> 4 \,\hbox {months}$	$< 4 \,\hbox {months}$	$> 4 \,\hbox {months}$
*N*	40	111	27	186	40	111	27	186
# items	7	7	7	7	8	8	8	8
Maximum Score	7	7	7	7	8	8	8	8
Mean score	4.99	5.77	5.98	5.62	5.60	6.42	6.39	6.20
Percentage	71.3	82.4	85.4	80.3	70.0	80.2	79.9	77.5

**Table 6:** Factors affecting the feasibility and acceptability of BFP

Parameters	Rural ($\hbox {N} = 111$)				Urban ($\hbox {N} = 186$)			
	Feasibility		Acceptability		Feasibility		Acceptability	
	OR	95% CI	OR	95% CI	OR	95% CI	OR	95% CI
Age								
10–19 years	.551	.198–1.531	.769	.204–2.898	.326	.029–3.668	1.885	.775–4.585
20–25 years	1.008	.156–4.969	.581	.233–3.671	3.991	.378–2.490	1.476	.420–3.871
$$>25 (ref)$$								
Education								
Lower primary school	1.315	.114–5.203	1.085	.795–3.807	–	–	–	–
Upper primary school	.596	.178–2.144	1.294	.693–4.936	–	–	–	–
High school	.923	.683–1.248	.735	.421–1.283	1.374	.269–2.002	.498	.220–1.127
Pre-degree/higher secondary school	1.277	.736–4.556	1.803	.161–4.399	2.061	.327–4.158	1.534	.549–3.783
College (ref)								
Profession								
Working professional	–	–	–	–	.792	.287–3.484	1.298	.249–2.058
Student	.924	.108–2.065	1.656	.631–4.895	.369	.164–3.242	1.461	.446–3.570
Homemaker	1.162	.574–2.269	1.238	.211–2.292	1.347	.820–369	.885	.433–3.780
Farmer (ref)								
Money spent every month								
More than ₹100	.703	.048–2.211	1.612	.858–3.156	.674	.066–3.671	.794	.470–3.483
₹50–₹100	.500	.242–1.000	1.713	.322–3.911	1.342	.989–2.402	.990	.701–2.342
$< 50$	1.233*	1.083–3.248	1.422*	1.203–3.748	.840	.294–2.420	1.442	.312–2.330
Using cloth pad and no expense (ref)								

*OR* odds ratio, *CI* confidence interval

*Significant at $$\textit{p}< 0.05$$l

### Acceptability of BFP

Focused studies on women’s perception of products available to them are limited [8]. The acceptability of BFP was assessed by analyzing the feedback received from both participant groups through the survey instrument (Table 4). Since almost all of the urban participants (96%) and 62% of the rural participants had used disposable sanitary pads prior to using BFP, they evaluated BFP’s performance against their prior choices. The factors used to assess acceptability included: preference of sanitary products, recommendation of BFP to others, comfort and ease of use and re-use of BFP, ease of washing and cleaning, influence and impact of BFP on other household members, preference on continued use of BFP and reasons for preferring BFP. When asked about their preferences of sanitary products, a majority of rural users i.e. 62.2% and a minority or urban users i.e. 22.6% were very convinced of switching to using only BFPs. On the other hand, approximately 52.2% urban women preferred to use a combination of both disposable pads and BFP. This suggests that while women were comfortable using BFP as part of their MHM, they preferred a phased transition into BFP.

With regard to recommending BFP to others, nearly 78% of urban participants and 64% of rural participants said they were likely to recommend it to their peers. Both groups either agreed or strongly agreed (93.7–93.9%) that BFPs were comfortable to use and reuse. Nearly 96% of rural participants and 51% of urban participants found them easy to wash and clean too. With regard to the influence of their usage of BFP on other household members, 60.4% of rural participants and 45.7% of urban participants indicated that other women in their family felt inclined to switch to BFP after sharing of their personal experiences. Up to 88% of rural participants and 74% of urban participants indicated they would continue to use BFP in their MHM routines.

Delving into the motivational factors for their preferences, we allowed participants to pick multiple answers for Q21 and Q22. While most users found the BFPs easy to use, clean, and presenting minimal leakage, the most popular reason for wanting to switch to BFP was its eco-friendliness and affordability. When asked about the primary reasons for their recommendation of BFPs to others, most respondents reported that environmental concern (64.5–78%) and health concerns were their predominant reasons. Additionally, monthly expenditure also mattered to the rural participants (52.3%).

### Effect of socio-demographics on feasibility and acceptability

The mean score and the percentage of feasibility and acceptability of rural and urban participants are shown in Table 5. These were based on the scoring rubric of 0, 0.5 or 1 assigned to responses that were negative, partially positive and fully positive respectively. The feasibility was found to be 82.4% (mean score = 5.77) and 80.3% (mean score = 5.62) amongst rural and urban participants. Similarly, the acceptability was found to be 80.2% (mean score = 6.42) and 77.5% (mean score = 6.20) amongst rural and urban participants respectively. To understand the feasibility and acceptability of participants that had used BFP for less than 4 months, we calculated their scores and compared them with those that had used BFP more than 4 months. The score and percentage of feasibility and acceptability of BFP are tabulated in Table 5. The results suggest high feasibility (rural: 71.3%, mean score $= 4.99$; urban: 85.4%, mean score $= 5.08$) and acceptability (rural: 70.0%, mean score $= 5.60$; urban: 79.9%, mean score $= 6.39$) amongst those who used BFP for less than 4 months as well.

The binomial logistic regression results of feasibility and acceptability of rural and urban participants (those who have used BFP more than 4 months) are tabulated in Table 6. The values of the model statistics like Omnibus tests, Hosmer and Lemeshow’s goodness-of-fit test, $-2$ log likelihood (-2LL) value of the model, Cox and Snell $R^2$ and Nagelkerke $R^2$ was checked and suggest good fitting by the model [53]. The results suggest that, age, prior education and profession of participants does not significantly affect the feasibility and acceptability of both groups. However, the cost of the product affects feasibility ($\hbox {OR} =1.233$; 95% CI = 1.083–3.248) and acceptability ($\hbox {OR}= 1.422$; 95% CI = 1.203–3.748) with respect to rural participants.

We also requested feedback from women who did not adopt BFP. Their feedback included heavy menstrual bleeding causing diffidence in switching from their current choices, lack of personal commitment to wash or reuse BFPs and insensitivity to environmental pollution from disposable non-biodegradable pads.

## Discussion

In the study conducted among 111 rural and 186 urban women, the feasibility and acceptability of a novel product (BFP), assessed from various user and environmental perspectives, was found to be 82.4% (mean score $= 5.77$) & 80.3% (mean score $= 5.63$) and 80.2% (mean score $= 6.42$) and 77.5% (mean score $= 6.20$) respectively (Table 5). These high levels of satisfaction indicate the viability of environmentally sustainable options for menstrual hygiene. In similar feasibility studies on reusable and eco-friendly alternatives such as cloth pads and menstrual cups leakage, comfort, availability and cost were characterized as key factors [54, 55] driving women’s preferences. Other studies raised concerns related to safety, hygiene, and insertion [56] as deterrents impeding their adoption [4, 55]. Reusable pads, however, allow greater flexibility in terms of use and reuse and are economically and environmentally more sustainable in the long term [57]. During strict quarantine brought about by COVID-19, re-usability of menstrual products like the BFP met women’s needs and alleviated menstrual poverty [28]. Yet, studies on feasibility and acceptability of such natural fiber based, reusable menstrual products were not available after numerous literature searches and hence were the focus of this study.

BFPs are sustainable alternatives due to their abundant availability, suitable mechanical properties and ease of production and manufacturing [4, 58–61]. Results from this work address key concerns with reusable menstrual products [54, 62] i.e. duration of single use, leakage, ease of wash and drying in pad maintenance. The user perceptions and pressure squeeze tests displayed satisfactory performance of BFP amongst both rural and urban participants. Study results were overwhelmingly positive despite prior studies suggesting that even minor usage inconveniences can dampen users’ initial enthusiasm and potentially cause product discontinuance [63].

Characterization of microbial load in a few selected samples based on monitoring of *S. aureus*, *Candida albicans*, and other pathogens indicated that the microbial load does not increase with repeated usage over 36 cycles. Literature confirms that used pads, when washed with detergent [64] and dried in sunlight [65], are safe to reuse, proving that improper maintenance causes commonly reported issues with reusable pads. While these results are preliminary, they corroborate with findings of similar studies on reusable menstrual cups that determined no severe pathogenic infections such as Candidiasis, Bacterial vaginosis, Cervicitis in most women after reuse [66]. However, conclusive data on the limits of BFP reuse without compromising safety will require an extensive investigation with larger groups of women.

A key aspect of feasibility of emerging menstrual product alternatives relates to its carbon footprint. Life cycle assessments of menstrual options [30, 49] show the spectrum of impact women’s choices have on their own health as well as the environment. A conventional pad with 10g of plastic [49] can take several centuries to decompose [67] in comparison to a BFP expected to take approximately 6 months to degrade [58]. Comparison of carbon emissions from disposable and BFPs showed relatively high differentials between them that are further enhanced when the latter is used as a reusable option.

Improper MHM, especially amongst rural women, is largely attributed to limited availability and cost-prohibitive menstrual products [20, 49, 54]. Approximately (22.5%) of our rural participants used makeshift materials and 60.4% of them spent less than 50 rupees only (US$ 0.65) per month. Provision of low cost disposable pads is seen as the solution to manage MHM [49] although women continue to have unmet needs [68]. Cost and affordability did emerge as predominant feasibility ($$\hbox {OR} =1.233$$; 95% CI = 1.083–3.248) and acceptability ($$\hbox {OR}= 1.422$$; 95% CI = 1.203–3.748) indicators based on the regression analysis done amongst the rural participants.

Analyzing factors contributing to acceptability, product preference, recommendation to others, comfort and ease of use and reuse, ease of washing and cleaning, others in the family wanting to switch to BFP and continuance of BFP use were found to be significant predictors (Table 4). These outcomes are consistent with other research on acceptability of menstrual material [56]. Although peer studies report that women resist making changes to absorbent choices made during menarche [69], surprisingly, our study found that age was not a significant predictor of acceptance (Table 6). Instead, lack of exposure to best practices is a key reason for women’s poor MHM choices [55]. The high acceptability result accedes with other studies that indicate experimental product provision alone for individual-intervention is ineffective [70] but supplementing users additionally with orientation on benefits, usage and resulting impact can enhance uptake of a new intervention [56, 63].

Women’s choice of materials to meet menstrual health needs may change depending on their contexts, needs, and preferences [5]. Design of natural products that provide choice, convenience and safety in addition to exposing women to benefits from trade-offs [4] are important directions to pursue in order to bring natural and reusable menstrual materials into mainstream MHM.

## Conclusion

Menstrual products should not only satisfy women’s MHM needs but also be affordable as it is a basic human right. The novel BFP holds out much promise in spite of the fact that maintenance methods of the BFP were new and possibly different from the prior practices of the participants. Our findings indicate that natural fiber based pads are a feasible alternative to disposable sanitary products. Pandemic era experiences and fears have temporarily profited reusable menstrual product businesses, but for their long-term survival in the market, rigorous studies need to be conducted.Specifically, repeated cross-sectional surveys and larger studies are required to establish BFP as a safe alternative to manage MHM. The strategy of provision of knowledge and orientation of benefits and trade-offs are critical to adoption of environmentally favorable products such as BFP.

## Supplementary Information

**Additional file 1.** Selected survey questions and their respective predictors.

## Data Availability

The data that support the findings of this study are available from the corresponding author upon reasonable request.
