# Supplementary material for: A novel banana fiber pad for menstrual hygiene in India: a feasibility and acceptability study
Source: BMC Womens Health. 2021 Mar 26;21:129. doi: 10.1186/s12905-021-01265-w (PMC7995390; doi:10.1186/s12905-021-01265-w)
Supplement: Supplementary file 1 — Additional file 1. Selected survey questions and their respective predictors. [file 12905_2021_1265_MOESM1_ESM.pdf]

A Novel Banana Fiber Pad for Menstrual Hygiene in India: A Feasibility and Acceptability study

Krishnashree Achuthan[1*], Sharanya Muthupalani[1], Vysakh Kani Kolil[1], Anju Bist[1], Krishna Sreesuthan[1], Aswathy Sreedevi[2]

[1]Amrita School of Engineering, Amrita Vishwa Vidyapeetham, Amritapuri, Kollam, 690 525, India
[2]Department of Community Medicine, Amrita Institute of Medical Sciences and Research Centre, Amrita Vishwa Vidyapeetham, Ernakulam, 682 041, India

Survey Questions

Part I: Demography

1. **Age :**
2. **Education :**
3. **Profession :**
4. **Which of these are you familiar with using? (Please tick all that apply)**

   ☐ Cloth absorbents that you can wash and use again.

   ☐ Disposable sanitary pad

   ☐ Menstrual Cup

   ☐ Tampons

   ☐ Others …………………………………

5. **How much money were you spending per month on sanitary products previously?**

   ☐ Less than ₹ 50

   ☐ ₹ 50- ₹ 100

   ☐ More than ₹ 100

   ☐ Cloth, so no expense

   ☐ Don’t know

6. **Are you the first in your family to use BFP (Banana Fibre based Menstrual Pad)?**

☐ Yes

☐ No

☐ Don’t know

7. **How long you are using BFP?**

☐ Less than 4 months

☐ More than 4 months

☐ More than 1 year

☐ More than 2 years

Part II: Characterization of Banana Fibre based Menstrual Pad (BFP)

A. **Feasibility:**

1. **Preferred place of usage**

☐ Any time

☐ At work/college/school

☐ At home only

☐ At night only

2. **Experience with leakage**

☐ Same as disposable pads

☐ Less leakage than disposable pads

☐ More leakage than disposable pads

☐ No leakage at all

3. Please indicate how long you use BFP without changing it?

- ☐ Less than 3 hours
- ☐ 3 - 4 hours
- ☐ 5 - 6 hours
- ☐ More than 6 hours

4. How much time does it take to wash BFP after use?

- ☐ Less than 1 minute
- ☐ 1 - 2 minutes
- ☐ More than 2 minutes

5. How do you wash your BFP?

- ☐ Brush wash
- ☐ Soak first in water and then wash by hands
- ☐ Machine wash
- ☐ Stone wash and later soak in disinfectant water for sometime

6. After washing, how long does it take for BFPs to dry?

- ☐ 1 - 3 hours
- ☐ 4 - 6 hours
- ☐ More than 6 hours
- ☐ One day
- ☐ Don’t know

7. Where do you dry your BFPs?

- ☐ In my room / bathroom
- ☐ In the open with other clothes, but I cover it with a towel
- ☐ In the open far from rest of the cloths
- ☐ In the drying machine

B. Acceptability:

1. Which of the following is true of your current absorbent choice?

- ☐ Both disposable pads and BFP
- ☐ BFP 80% of the time
- ☐ Menstrual cup and BFP
- ☐ Only BFP
- ☐ BFP and another brand of reusable pads

2. How likely are you to recommend BFP to other women?

- ☐ Very likely
- ☐ Likely
- ☐ Unlikely
- ☐ Very unlikely

3. The BFPs are comfortable and easy to use and reuse

- ☐ Strongly agree
- ☐ Agree
- ☐ Neutral
- ☐ Disagree
- ☐ Strongly disagree

4. BFPs are easy to wash and clean

- ☐ Strongly Agree
- ☐ Agree
- ☐ Neutral
- ☐ Disagree
- ☐ Strongly disagree

5. **Are others in the family now wanting to make switch to BFP?**

   - ☐ Yes
   - ☐ No
   - ☐ Don’t know

6. **How likely you will continue using the BFP?**

   - ☐ Very likely
   - ☐ Likely
   - ☐ Unlikely
   - ☐ Very unlikely

7. **Reason for preferring BFP (Please tick all that apply)**

   - ☐ Easy to handle
   - ☐ Absorbs better
   - ☐ Feels clean
   - ☐ Economical
   - ☐ Eco-friendliness
   - ☐ I feel traditional
   - ☐ I feel modern

8. **Reason for recommending BFP (Please tick all that apply)**

   - ☐ Concern for the environment
   - ☐ Concern for the health
   - ☐ Money spent every month
